# First report on mycetoma in Turkana County—North-western Kenya

**DOI:** 10.1371/journal.pntd.0011327

**Published:** 2023-08-14

**Authors:** María Francisca Colom, Consuelo Ferrer, John Lochuke Ekai, David Ferrández, Laura Ramírez, Noelia Gómez-Sánchez, Simion Leting, Carmen Hernández

**Affiliations:** 1 Laboratory of Medical Mycology, Universidad Miguel Hernández, Avenida Santiago Ramón y Cajal s/n, Edificio Muhammad Al Shafra, Sant Joan d’Alacant, Spain; 2 Alicante Institute for Health and Biomedical Research (ISABIAL), Alicante, Spain; 3 NGO Medical direction, Cirugía en Turkana (*Surgery in Turkana*), Madrid, Spain; 4 Medical Diagnosis Laboratory, Lodwar County and Referral Hospital, Turkana, Kenya; 5 San Carlos University Hospital, Madrid, Spain; Albert Einstein College of Medicine, UNITED STATES

## Abstract

Mycetoma is one of the six Neglected Tropical Diseases that are prevalent in Turkana County (northwest Kenya). The aim of the study was to estimate the prevalence of mycetoma in the county, as well as to describe the main causative agents involved in the disease using methods affordable locally. Based on the data collected by the team of cooperative medicine *Cirugia en Turkana* (Surgery in Turkana), a specific study for mycetoma was started during the 16^th^ humanitarian medicine campaign in February 2019. Patients with suspected mycetoma were studied at the Lodwar County Referral Hospital (LCRH). After informing the patient and getting their consent, the lesions were examined and sampled (mainly by biopsy) and clinical data were recorded. Samples were washed in sterile saline solution and cut in fragments. Some of these were inoculated on Sabouraud Dextrose Agar, Malt Extract Agar, and diluted Nutrient Agar plates. One fragment of each sample was used for DNA extraction. The DNA and the rest of the fragments of samples were kept at -20°C. All cultures were incubated at room temperature at the LCRH laboratory. The DNA obtained from clinical samples was submitted to PCR amplification of the ITS-5.8S and the V4-V5 16S rRNA gene region, for the detection and identification of fungi and bacteria respectively.

From February 2019 till February 2022, 60 patients were studied. Most of them were men (43, 74,1%) between 13 and 78 y.o. (mean age 37). Half of the patients were herdsmen but, among women 40% (6) were housewives and 26.7% (4) charcoal burners. Lesions were mainly located at the feet (87.9%) and most of the patients (54; 93.1%) reported discharge of grains in the exudate, being 27 (46.6%) yellow or pale colored and 19 (32.8%) of them dark grains.

Culture of clinical samples yielded 35 fungal and bacterial putative causative agents. Culture and molecular methods allowed the identification of a total of 21 causative agents of mycetoma (39.6% of cases studied). Most of them (17) corresponded to fungi causing eumycetoma (80.9%) being the most prevalent the genus *Madurella* (7; 41.2%), with two species involved (*M*. *mycetomatis* and *M*. *fahalii*), followed by *Aspergillus* (2; 11.8%). Other minority genera detected were *Cladosporium*, *Fusarium*, *Acremonium*, *Penicillium*, and *Trichophyton* (5.9% each of them). Actinobacteria were detected in 19.1% of samples, but only *Streptomyces somaliensis* was identified as a known agent of mycetoma, the rest being actinobacteria not previously described as causative agents of the disease, such as *Cellulosimicrobium cellulans* detected in two of the patients.

Although Kenya is geographically located in the mycetoma belt, to our knowledge this is the first report on mycetoma in this country from 1973, and the first one for Turkana County.

## Author summary

In this paper we describe the medical situation of people affected by mycetoma in Turkana County, (north-west Kenya). This is a neglected disease, suffered by the poorest people in the world due to lack of education and access to health services. In Turkana there is a high incidence of this disease, especially among young men working in the fields, where they get infected by fungi or bacteria in the soil or on plants and thorns. The lack of nearby health care means that these patients’ injuries develop into severe disability and even death. The study of the tissues of patients detecting and studying the microorganisms involved, led to a more specific knowledge about the disease in this area of the world. Our work aims to contribute to the global knowledge of mycetoma to favor the implementation of means for the diagnosis and treatment of this problem, which will allow a better management and the implementation of satisfactory medical solutions.

## Introduction

Mycetoma is a devastating chronic infection of the subcutaneous tissue that spreads slowly locally, affecting skin and deep structures. It causes deformity, destruction, and disability, especially in advanced stages [1,2]. There are two types of mycetoma: actinomycetoma, caused by bacteria and predominant in Latin America; and eumycetoma, caused by fungi and predominant in Southeast Asia and Africa, in the semi-arid areas of Sudan, Ethiopia, Somalia, Chad, Niger and Mali, where it accounts for more than 85% of mycetoma cases reported so far [2,3]. The most frequent causative agent of actinomycetoma is *Nocardia brasiliensis*, an actinobacterium that responds well to antibiotic treatment. For eumycetoma, the fungus most frequently implicated is *Madurella mycetomatis*, although a wide variety of fungal species have been described [2,3]. The location of the disease is mainly the feet (>75%), followed by the hands and other locations such as legs, arms, trunk, neck, or head [2–4].

The disease is thought to be acquired by small trauma leading to the inoculation of the causative agents into exposed areas of the skin [4,5]. The disease begins as a painless lesion and slow-growing indurated subcutaneous nodules, later leading to the formation of large tumors that develop into necrotic abscesses. The tumors ooze sero-bloody or purulent content with macroscopic grains through fistulous tracts. These grains may be dark (black, brown or greenish), pale (yellow or white), or even red in color. The tumor may extend to adjacent structures such as muscle, bone, and lymphatic vessels [2–5]. In the context of the poorest regions of Africa such as Turkana, when most patients get health assistance the disease is already very advanced, so many cases end in surgical amputation and disability [6].

The population most frequently affected live in areas with poor health resources in the region of the world known as the ’mycetoma belt’, which includes tropical and subtropical areas of the Americas, Africa, and Asia with low rainfall [2]. In 2016, mycetoma was recognized as a Neglected Tropical Disease by the World Health Organization (WHO) [7]. Furthermore, its causative agents have recently been included in the WHO Fungal Priority Pathogens List of dangerous fungi for humans (FPPL- WHO, 2022) [8].

Most diagnosed cases of mycetoma are reported from Mexico and Sudan [9–11], followed by several countries in the "mycetoma belt" such as Senegal, Somalia, Niger, and others close to or even bordering Kenya, such as Uganda [12–14], although very scarce information has been reported from Kenya so far [12,15].

*Cirugía en Turkana* (Surgery in Turkana) is an NGO of surgeons and other medical specialists from Spain that has been carrying out annual humanitarian surgical cooperation campaigns in Turkana County (Kenya) since 2004. Based on the data collected by this NGO, we knew that the estimated prevalence of mycetoma was very high in the region. In fact, between 2013 and 2018, 75 cases of mycetoma (10% of the patients seen) were clinically diagnosed during the surgical campaigns [16]. As no data about the presence of the disease in the county had been published before, the NGO decided to include a mycologist in their team to start an epidemiological study of mycetoma in Turkana. An education program for the health workers and the population, which focused on the prevention and early diagnosis of the disease was also set up. From 1973 to date, there are no published data on the incidence of mycetoma in Kenya, so this study may be the first update on the epidemiology of the disease in this country and the first report on Turkana County.

The aim of the work was twofold, on the one hand, to estimate on the epidemiology of mycetoma in Turkana County, and identify the causative agents most frequently implicated in these lesions. On the other hand, to train local health professionals to implement a method of early detection and clinical and laboratory diagnosis at least at the level of distinguishing actinobacteria versus fungi. This would allow them to successfully manage the problem with the available resources and at an affordable cost.

## Materials and methods

### Ethics statement

A cross-sectional study was carried out from 2019 to 2022, at the time of the respective surgical campaigns of *Cirugia in Turkana* in the county. The study was conducted according to the guidelines of the Declaration of Helsinki and approved by the Institutional Review Board of University Miguel Hernandez (ref: DPV.MCV.01.20) and by the Ethics Committee of the following institutions: San Carlos University Hospital of Madrid (ref. 19-038-E), General University Hospital of Alicante (ref: PI2020/118), and Alicante Institute for Health and Biomedical Research (ISABIAL) (ref: 200196), as well as by the Director and the Chief Executive Officer of the Lodwar County and Referral Hospital (LCRH) in Turkana (Kenya). Written Informed consent was obtained from all subjects involved in the study.

### Patient selection

The study subjects were the Turkana population served by the LCRH. Patients were selected by local health personnel working in coordination with the Surgery team.

Almost all cases were studied during the time of the NGO’s surgical campaigns. The inclusion criteria were patients with suspected mycetoma lesions who gave their written (signed) consent after receiving information about the study. Swahili and Turkana interpreters and translators were used to ensure understanding of the informed consent form. In terms of exclusion criteria, no samples were taken from patients who did not understand the interpreter’s explanations or who refused to give consent.

### Demographic and clinical data

Demographic and clinical data were collected, as well as photographs of the lesions. The variables to be considered were age, sex, place of residence, main activity (agriculture, livestock, crafts, etc.), and others such as travels outside the county. In terms of clinical data, location and size of the lesion, secretion with grains, color of the grains and time of development of the disease were recorded (S1 Doc).

The size of the lesions and its relationship with the type of mycetoma, the color of the grains, and the length of the disease evolution (according to what was declared by the patient), were analyzed by 1-factor ANOVA with 4 levels (≤2 years, 3–5 years, 6 to 10 years and ≥10 years of evolution). Subsequently, Shapiro-Wilk normality test and Levene homogeneity test of variance were applied to check the robustness of ANOVA [17].

### Sample collection

Samples mainly consisted of biopsies, exudates, and complete surgical specimens (mycetoma mass). Biopsies and surgical specimens were obtained by surgeons under local anesthesia in the operating theatre. Exudates were collected by the healthcare professionals in the consultation room or in the laboratory with sterile swabs. All of them were immediately transported to the LCRH diagnostic laboratory.

### Processing of clinical samples

#### Macroscopic and microscopic observation

Clinical samples were subjected to detailed macroscopic and microscopic observation. Imprint of biopsies and smear of exudates were prepared for microscopic observation under a conventional light microscope with lactophenol blue and Gram stain. As far as possible, microphotographs were taken with the aid of a mobile phone camera adapted to the ocular. Subsequently, grains were washed with saline solution and biopsies were cut into small fragments before processing for culture and DNA extraction. Swabs were washed in sterile saline solution that was transferred into two sterile microtubes [18,19]. One of the microtubes and a part of solid samples were kept at -40°C in a freezer (Thermo Fisher Scientific 7240V).

#### Culture

A portion of the grains, biopsy fragments and/or 100 μL of the exudates were seeded on Sabouraud Dextrose Agar (SDA), Malt Extract Agar (MEA) and diluted Nutrient Agar (dNA) plates [18–21]. All cultures were incubated at room temperature and observed daily. After growth of microorganisms was detected, it was processed for microscopic observation with lactophenol blue and Gram stain and corresponding microphotographs were obtained. When necessary (growth of more than one type of colonies), isolation of the different microorganisms was performed in the same culture media. Cells of all isolates were kept in sterile water at 4°C and in skimmed milk at -20 or -40°C for further processing [22].

#### Extraction of nucleic acids

Samples kept at -20°C were used for DNA extraction. To obtain microbial DNA, all samples underwent the *Instagene Matrix* DNA extraction kit. Some of them were additionally subjected to the *DNeasy Plant MiniKit* (Qiagen). For some of the samples, none of these procedures led to the extraction of the DNA of the mycetoma causative agent; these difficulties were previously mentioned by W. van de Sande and her group [23–25]. Therefore, some additional assays were performed with the different options suggested for the NZY Plant/Fungi gDNA Isolation kit (NZYTech, Lda.–Genes & Enzymes. Lisbon. Portugal). Based on these results, the procedure was enlarged with previous dehydration of the samples with ethanol, followed by homogenization with glass beads.

#### Phenotypic study of the isolates

- **Morphology**. Macroscopy features of the colony growth in the different culture media was considered for identification. This aspect was especially important in the case of filamentous fungi. For them, speed of growth, color of the front and reverse of the colonies, as well as their texture, was carefully observed and recorded at different incubation times in SDA and MEA. Microscopic appearance of the cells obtained from cultures was analyzed by conventional light microscopy at different magnifications. As mentioned before, Gram stain and lactophenol blue were used for bacteria and fungi, respectively [26].
- **Physiological and biochemical tests**. For the characterization of bacteria, tests available at the LCRH were performed following simple identification algorithms. These included gram stain, peroxidase, oxidase, and coagulase production. Additionally, cellulolytic activity was added for characterization of *Cellullosimicrobium cellulans* [19,27,28].

#### Molecular study of the isolates

- DNA extraction from fungal and bacterial isolates was mainly performed using the *Instagene Matrix DNA extraction kit* (Bio-Rad Laboratories, Inc). As mentioned for the DNA extraction from the clinical samples, the standard methods failed for some fungal isolates, so the amplified NZYtech protocol was applied in such cases.
- PCR amplification. Extracted DNA was submitted to amplification of the ITS1-2 fragment of the ribosomal region of fungi and V4-V5 of the 16S rRNA of bacteria by simple one-round PCR [29,30]. PCR products were separated by electrophoresis in agarose gel (1.8%) using an ENDURO Gel XL electrophoresis system (Labnet). After electrophoresis, presence of DNA bands of expected size was detected by visualization of the gels in a FastGene B/G LED Transilluminator (Nippon Genetics, Europe GmbH) in the LCRH and by BioRad Gel Doc XR- System (Bio-Rad Laboratories) in Spain.
- Molecular identification. PCR products were kept at -40°C and subsequently transported to the Medical Mycology Lab at University Miguel Hernandez (Alicante. Spain). For all the samples that yielded a positive result the DNA was cleaned (illustra ExoProStar Cytiva) and sent to a reference laboratory to obtain the sequence (Centro Inmunológico de Alicante; Eurofins Megalab-Madrid, and Macrogen -Madrid, depending on the time in which the study was carried out). For the molecular identification, sequences were compared with the ones in databases such as GenBank (NCBI) and Mycobank database (IMA/WFBI) for fungal identification, and GenBank (NCBI) for bacteria.

### Treatment of mycetoma

Treatment protocols were adopted according to literature [31,32] and the possibilities in Turkana. Surgical treatment was only indicated in small eumycetomas, [32] but drugs were necessary for all the cases. Given that mycetoma is a neglected tropical disease, most of the patients were poor or very poor. Kenya’s public healthcare system does not cover mycetoma treatments. On top of that, some of the drugs that have been proposed as the golden standard for treatment (such as itraconazole for eumycetoma) are not available in Turkana. Therefore, a decision was made to choose a treatment protocol that the research project and the NGO could afford to cover, and to use drugs that were available in Turkana (such as fluconazole), despite its known low efficacy on *Madurella mycetomatis* infections [33]. The treatment protocol was as follows: For small eumycetomas (less than 5 cm), surgical treatment was the first choice, removing the mass from soft tissues, with a subsequent prescription of fluconazole (200 mg/day) for two to three weeks to ensure efficacy against the disease. For larger eumycetomas (> 5 cm) or eumycetomas located in areas with difficult or risky surgical access, medical treatment with fluconazole was the first choice, with a 200 mg dose a day during four to six months. Afterwards, surgery could be considered for removing the remaining mass and, subsequently an additional period of medical treatment of one month was prescribed. For the actinomycetomas, medical treatment was the first choice. During the 2019 campaign, IM streptomycin (15 mg/Kg/day) combined with oral rifampicin (10 mg/kg/day) was prescribed for periods of 3 weeks to 6 months. From 2020, IM amikacin 15mg/Kg/day divided in two doses and oral cotrimoxazole (sulfamethoxazole 35 mg/kg/day and trimethoprim 7 mg/kg/day divided into three doses) for periods ranging from 1 to 6 months was prescribed [31].

### Training of health professionals

During the NGO campaigns, all the procedures described above for the obtention of samples and their subsequent study in the laboratory were performed in the presence of local workers that had been trained for this. In the inter-campaign periods, the contact between all members from the Spanish and the Kenyan sides, was kept through phone and internet whenever necessary for consultation.

## Results

### Population studied

During the three humanitarian campaigns carried out between February 2019 to February 2022, 60 cases of mycetoma were collected and selected to be treated and included in this study.

After careful clinical examination, two patients were considered as misdiagnosed and were discarded from the study. Another five patients gave their consent to be included in the study but did not consent sample retrieval. In addition, there were eight cases where two samples (biopsy and exudate) were obtained from the patients. Therefore, 58 patients were included in terms of clinical and demographic data and 61 samples corresponding to 53 patients were analyzed.

### Estimation of prevalence

Based on all the cases diagnosed and registered by the NGO between 2013 and 2022 on a population of 926,976 in the Turkana County [34], the prevalence of mycetoma can be estimated in 1.46 cases by 100,000 inhabitants per year. Based on the data corresponding to the 60 patients already seen, the distribution of the disease in the sub-counties shows a higher prevalence in Turkana Central and Loima, and the mean prevalence in number of cases per habitants and year is 1.33 (Table 1).

**Table 1:** Global prevalence of mycetoma in the county and its distribution by sub-counties. Population data from Kenya National Bureau of Statistics [33].

*SUB-COUNTY*	*CENTRAL T*	*NORTH T*	*WEST T*	*LOIMA*	*KIBISH*	*SOUTH T*	*EAST T*	*TOTAL / MEAN*
*TOTAL*	14	3	11	11	1	2	7	49
*SUB-COUNTY POPULATION*	185,305	65,218	239,627	107,795	36,769	153,736	138,526	926,976
*CASES / 100000 HAB*	7.6	4.6	4.6	10.2	2.7	1.3	5.1	5.3
***cases /10^5/year***	**1,9**	**1,15**	**1,15**	**2,55**	**0,68**	**0,36**	**1,28**	**1,33**

### Demographic and clinical variables

No gender data were recorded for one of the 58 patients. As for the others, 42 were male (73.41%) and 15 female (25.86%). They ranged in age from 13 to 78 years, and most of them under 31 (48.27%). The vast majority of patients were people who worked in the countryside (57.14%), i.e. shepherds, farmers and peasants, followed by salesmen and housewives among other professions. Regarding the type of footwear used, 48 (82,8%) patients confirmed the regular use of footwear, where 39 (81,3%) of them used very simple open shoes made of used tyres, 3 (7.7%) used closed shoes, 6 (15.4%) cases did not specify what type of shoes they had. In the group that did not regularly use footwear, five patients (50%) confirmed occasional use of footwear, and one (10%) declared not using footwear at all. There are 4 (6.9%) patients for whom there is no information on this variable.

**Fig 1:**
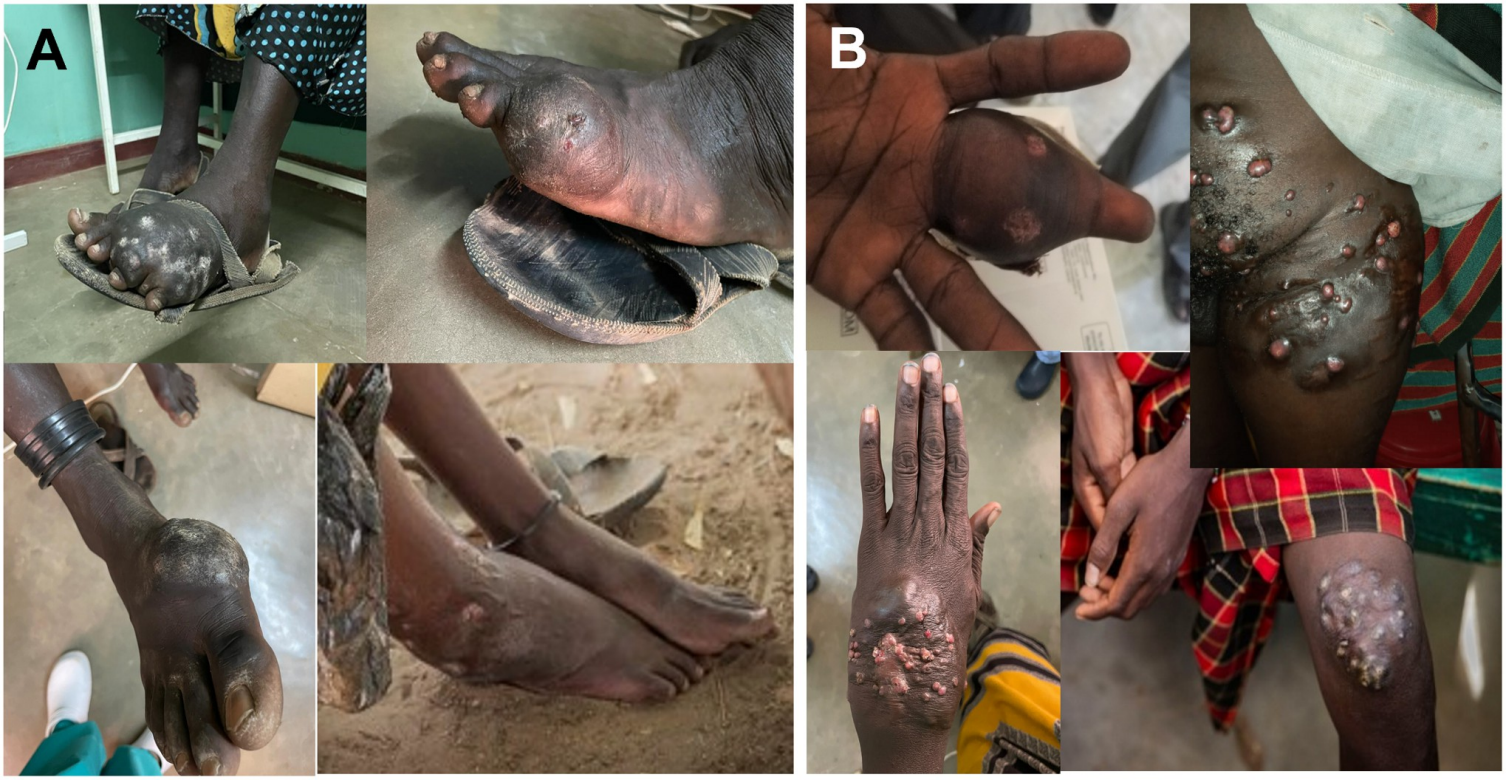
External aspect of mycetomas showing the characteristic painless tumor with sinuses. A) Different appearance of mycetoma lesions located on the feet B) Mycetoma lesions in other body locations.

**Fig 2:**
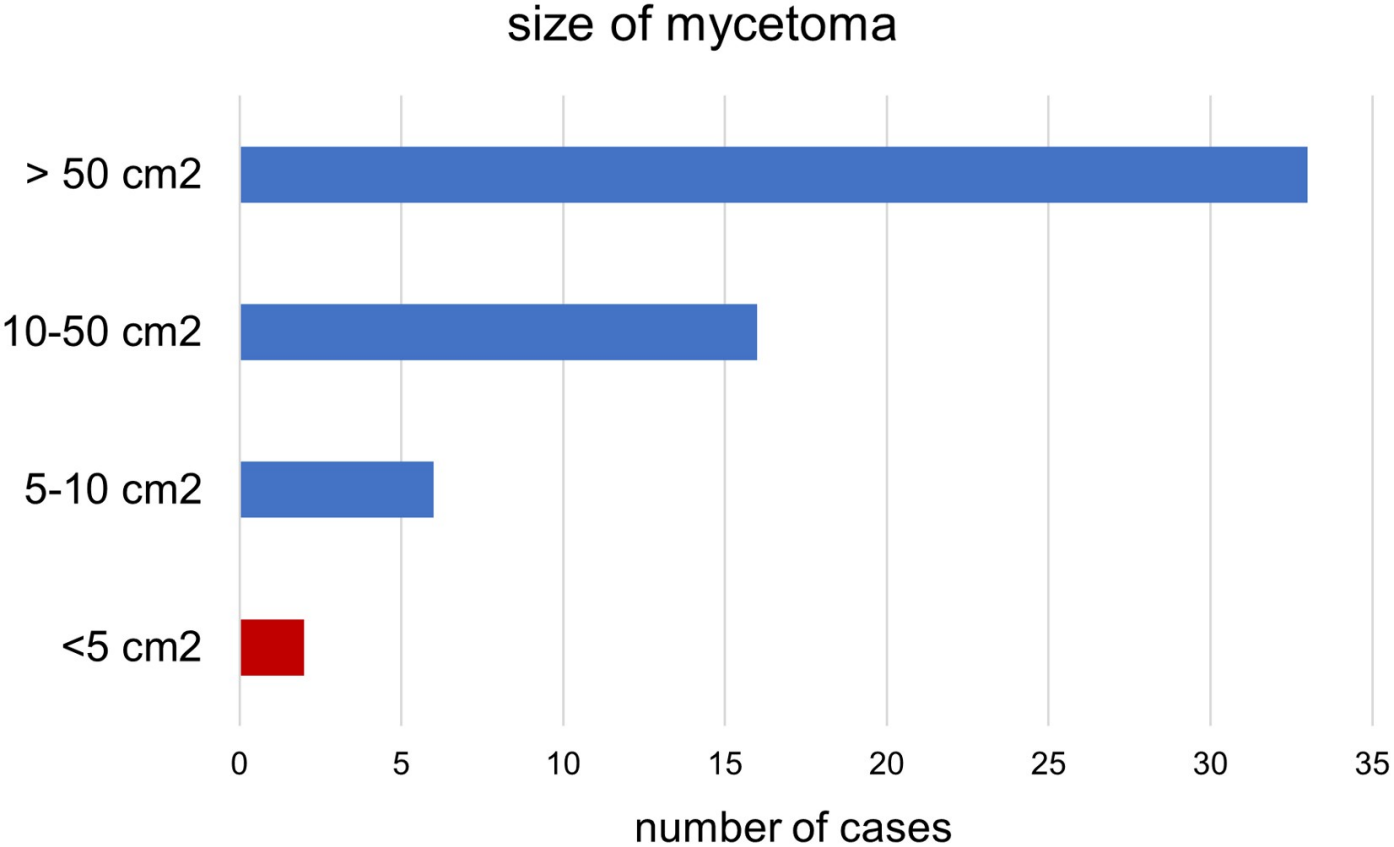
Size of mycetoma in square centimeters. Red bar represents mycetomas under 5 square centimeters, in which surgical removal is indicated as first choice for treatment.

Most of the lesions were located on the feet (87.9%), with a slight tendency towards the right foot (60.8%), although lesions were also observed in other areas such as hands (5.1%), legs (3.5%), and other areas (perineum, trunk, and ankles) (5.1%) (Table 2 and Fig 1). The time of evolution of the lesions varied from 1 to 37 years with a mean of 6 years. The size varied from lesions limited to an area a few square centimeters (2 cm^2^) to lesions that cover a large body area (Fig 2). Unique lesions as large as 20 x 15 cm have been observed in these patients. ANOVA analysis showed no significant relationship (p >0.05) between size of the lesions and the kind of mycetoma (p = 0.639), the color of the discharged grains (p = 0.888) and the time of evolution according to what was declared by the patient (p = 0.557) (S1 Fig).

**Table 2:** Location of mycetoma in the body area.

	FOOT	LEG	HAND	TRUNK	TOTAL
**TOTAL**	51	2	3	3	**59**
**RIGHT**	31	1	2	1	**35**
**LEFT**	15	1	1	2	**19**
**N/D**	5	0	0	0	**5**
	**87.9%**	**3.45%**	**5.17%**	**5.17%**	

**Fig 3:**
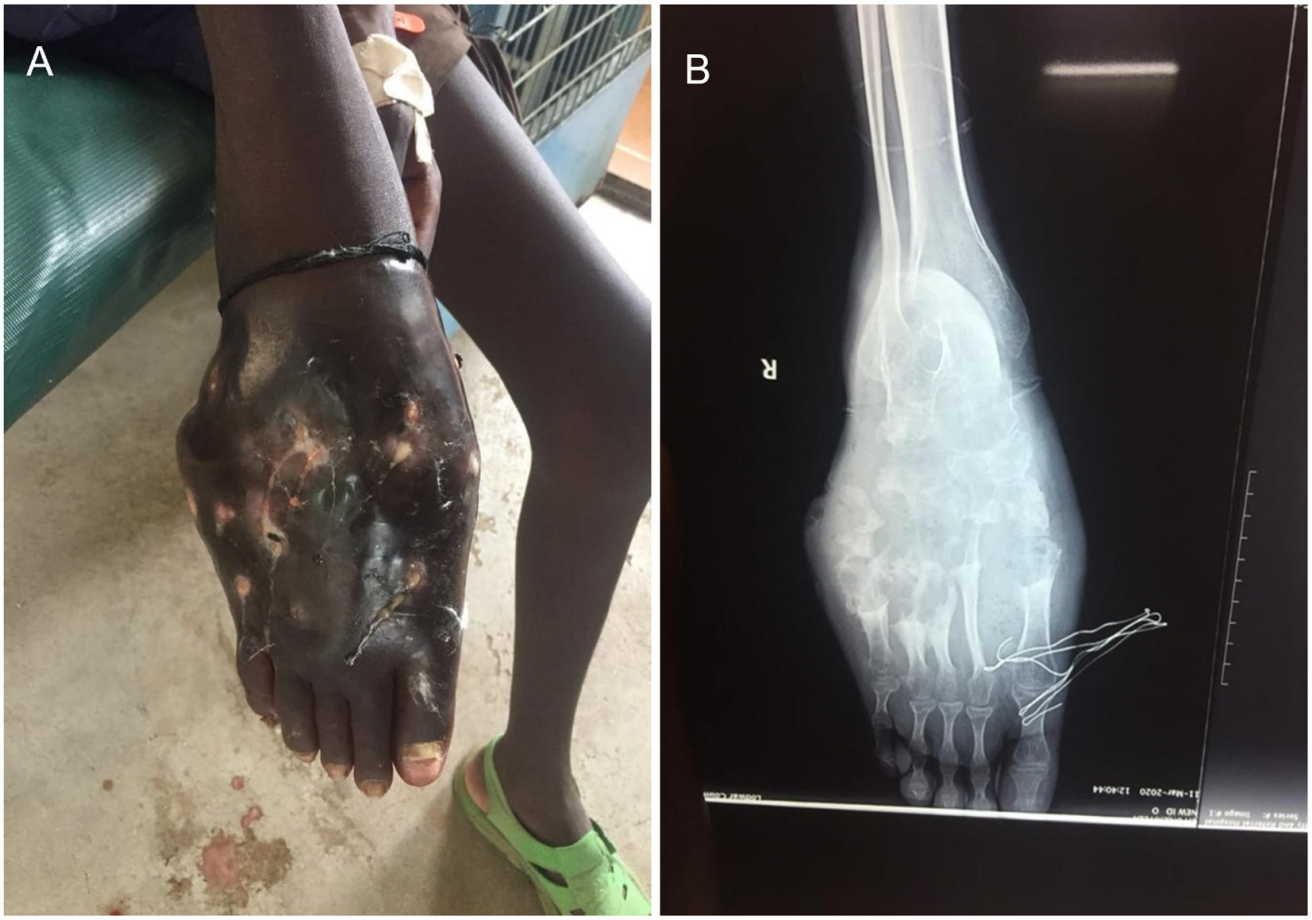
Large mycetoma affecting the right foot of a 15-year-old patient. A) Aspect of the foot at direct examination. B) Radiology of the same area showing the involvement of bones and deep tissues. The patient needed amputation to avoid the spreading of mycetoma in the lower limb.

Radiology and ultrasound were not always available and therefore, bone involvement was only evaluated in the cases with large lesions in which amputation was considered (Fig 3).

One of the key features of this infection is the secretion of grains in the exudate. Most of the patients (54; 93.1%) reported or showed discharge of different types of grains: 27 secreted yellow grains (46.6%), 19 had black grains (32.8%), 3 had white grains (6.9%) one patient reported greenish grains (1.7%), and another reported brownish ones (1.7%). These two were considered as dark grains and grouped together with the black ones. Two of the patients (3.45%) could not define the color of the oozing grains and for 4 patients (6.9%) the data was not available.

### Laboratory diagnostic results

#### Samples collected and studied

As mentioned before, 61 samples were collected from 53 patients. Most were biopsies (45, 73.779%), followed by exudate (6; 9.83%) and a minority were surgical specimens, grains and swabs.

#### Detection and identification of microbial agents by conventional phenotypic and molecular procedures

**Fig 4:**
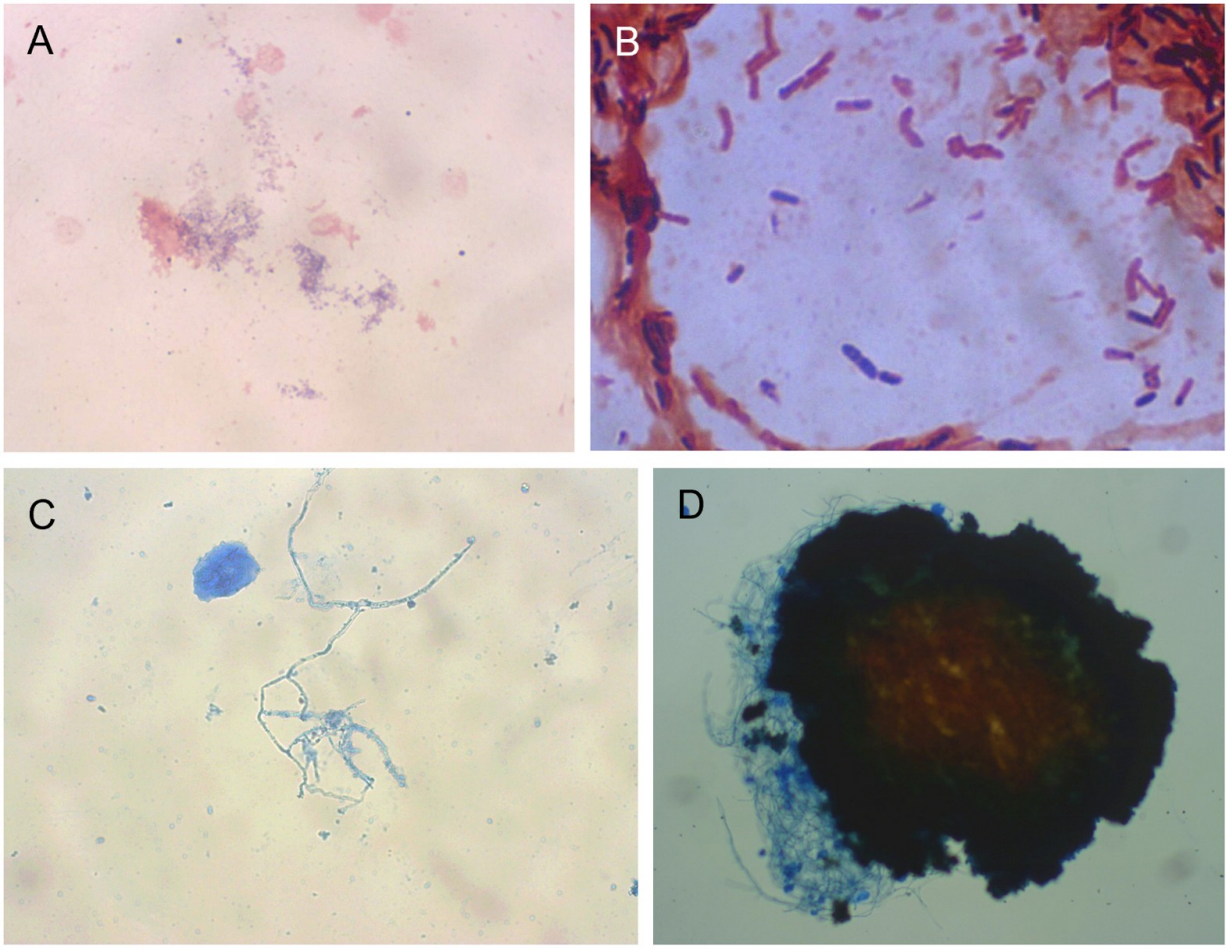
Microscopy of samples: A) Gram stain of the biopsy print from patient M05 showing thin grampositive rods (X400). B) Gram positive rods from the exudate of patient M08 (X1000). C: Lactophenol blue stain showing fungal filaments from the sample of patient M09 (X400). D) Microscopy of a black grain of *Madurella mycetomatis* from patient M07 after conventional treatment for DNA extraction (x100).

After samples collection, tissue imprint or exudate smears stained by Gram and/or lactophenol blue were observed under conventional optical light microscope. For eleven samples (18%), the presence of bacteria could be detected, and fungal structures were seen in eight of them (13.12%) (Fig 4). For most of the samples (42; 68.88%) the direct visualization was not informative.

**Fig 5:**
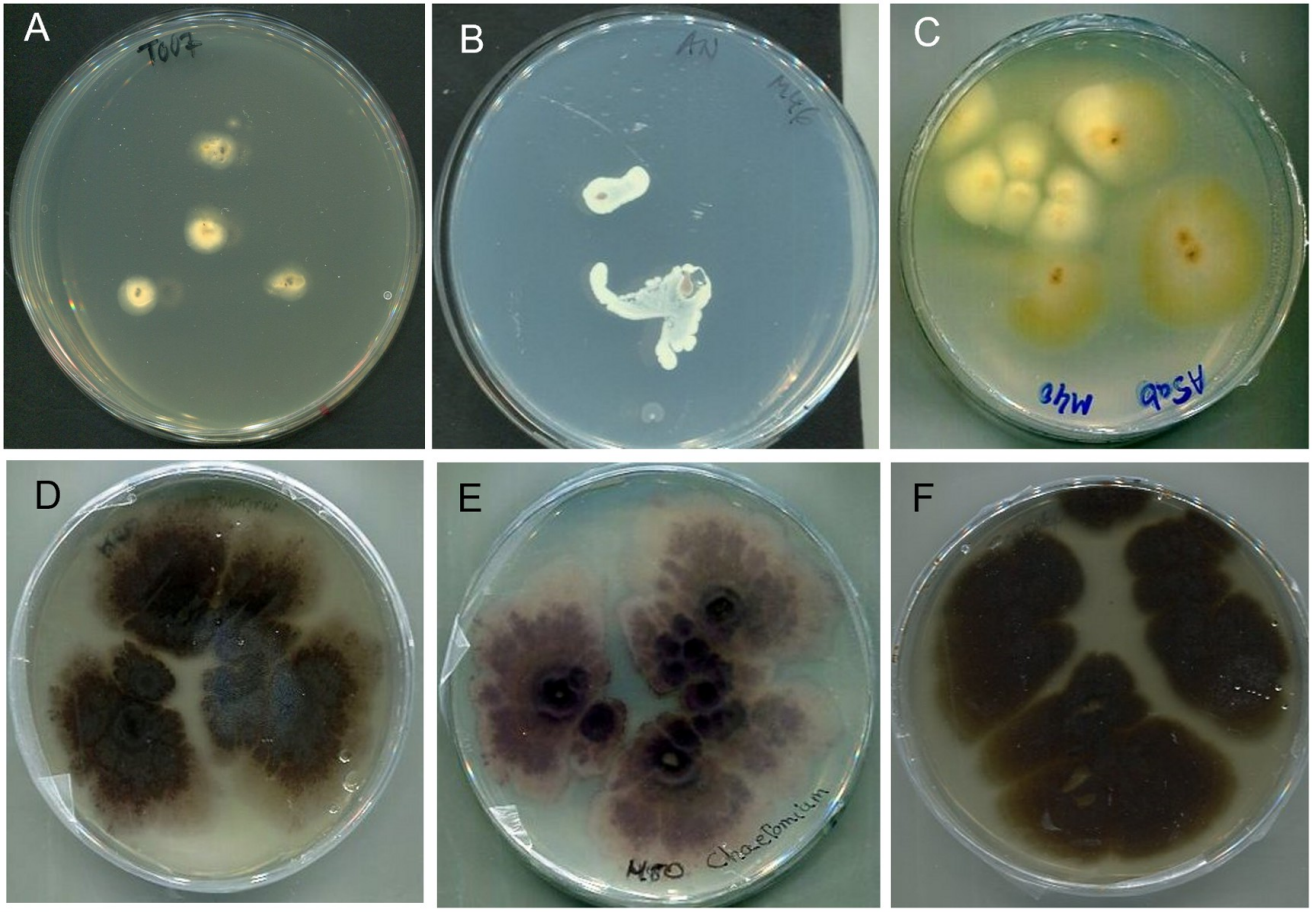
Growth of mycetoma agents from samples inoculated on SDA and dAN. Upper row: Aspect of the fungal and bacterial growth in the first days of incubation. A) Sample from patient M07 after five days); B) Samples from patient M046 after five days incubation. C) Samples from patient M040 after 10 days incubation on SDA. Lower row: macroscopic aspect of isolates obtained from samples of patients M040 and M050 after 3 weeks of incubation corresponding to species not previously described as causing mycetoma: D and E) *Subramaniula thielavioides* (front and back side) and F) *Macroventuria anomochaeta*.

61 samples were processed and cultured. Microbial growth was observed in 43 of them (70.5%) while the remaining 18 (29.5%) were negative in all media assayed. Among the positive, bacterial growth on nutrient agar was obtained from 27 samples (62.8%), mainly showing white or yellowish, mucous, or creamy colonies (Fig 5). Fungal growth was developed from 29 samples (67.4%) and corresponded to filamentous colonies of diverse morphology (Fig 5). Samples from 13 patients yielded both kinds of growth (30.2%).

Phenotypic study of the isolates led to the suspicion that several of them could be considered as bacterial and fungal contaminants from soil and skin. They corresponded to some grampositive cocci, like *Staphylococcus* spp, yeasts like *Candida* species and some filamentous fungi that appeared together with other fungi or bacteria that were more likely the cause of the mycetoma. For bacterial growth, 13 isolates (48.2%) of the total bacterial isolations corresponded to contaminants; for fungal growth, 10 isolates (34.5%) were also considered as contaminants (Table 3).

**Table 3:** Molecular Biology and Culture based identification of mycetoma causative agents.

Molecular Biology			Cultures		
Samples/DNA processed		61/42	Samples/positive cultures		61/43
ITS AMPLIFICATION		percentage	FUNGI		percentage
**positive**	**32**	**76.2%**	**positive**	**29**	**67.4%**
dirty sequences	4	12.5%	identified	19	65.5%
contaminant	15	46.9%	contaminant	10	34.5%
Mycetoma agent	8	25%	Mycetoma agent	9	31.03%
16S AMPLIFICATION			BACTERIA		
**positive**	**29**	**69.1%**	**positive**	**27**	**62.8%**
dirty sequences	5	17.2%	identified	15	55.6%
contaminant	20	69.0%	contaminant	13	48.2%
Mycetoma agent	4	13.8%	Mycetoma agent	2	7.4%

Molecular study of the samples detected the fungal target (ITS) in 32 samples (76.2%). The bacterial target (16S) was detected in 29 samples (69.1%). Thirteen (31%) samples showed positive results for both targets. The sequence of the amplicons allowed the identification of 23 fungal and 24 bacterial species. For nine of the amplicons obtained (21.4%) the sequences were not enough clean to be used for identification.

Among these, 20 (69%) 16S sequences and 15 (46.9%) of the ITS corresponded to species considered as contaminants from soil and skin (Table 3).

Cultures and molecular methods allowed the identification of the causative agent of mycetoma on 21 of sampled patients (39.62%), where the most prevalent genus was *Madurella* with two species involved: *Madurella mycetomatis* (4 cases) and *Madurella fahalii* (3 cases). *Aspergillus* species (2 cases), *Acremonium sclerotigenum*, *Cladosporium ramotenellum*, *Fusarium* sp, *Penicillium thomi*, *and Trichophyton interdigitale* (one case of each) were identified as minority agents of eumycetoma. For bacteria causing actinomycetoma, *Streptomyces somaliensis* was the only known actinomycetoma agent detected (one case).

**Fig 6:**
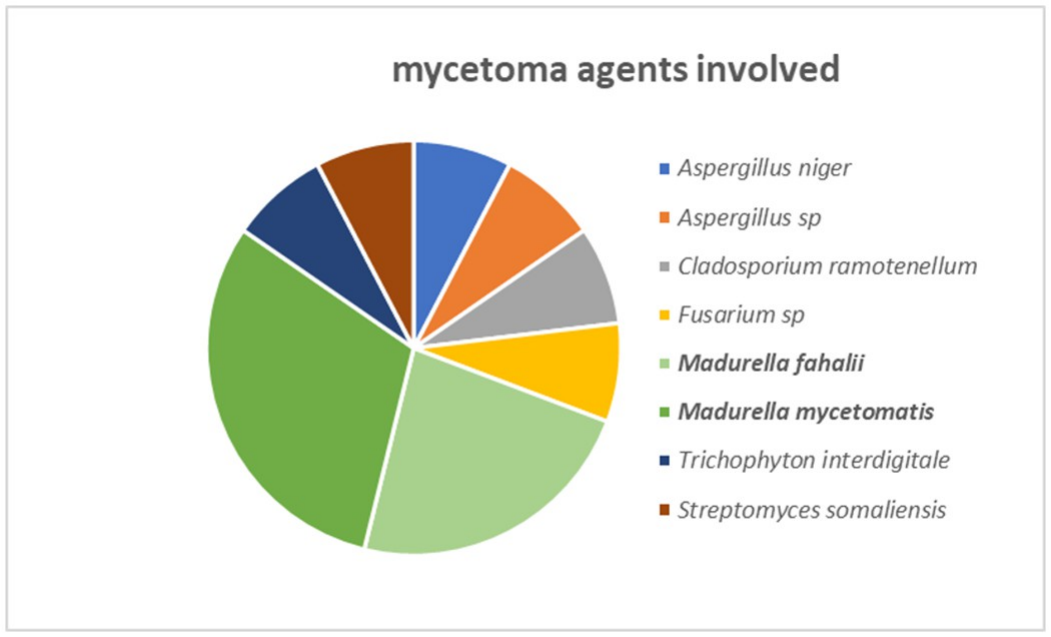
Fungal and bacterial mycetoma agents detected in the samples of the studied cases of mycetoma.

Other five species were isolated and identified from the samples but do not correspond to agents previously described as causing mycetoma. Three of these corresponded to fungal species: *Macroventuria anomochaeta*, *Subramaniula obscura*, *Subramaniula thielavioides* (one case of each), and two corresponded to bacteria: *Cellulosimicrobium cellulans* that was detected in two cases and *Corynebacterium mucifaciens* in one case. Additionally, seven more isolates were considered as contaminants of the samples, consisting in bacterial isolates of *Sphingomonas echinoides*, *Enterococcus malodoratus*, *Cutibacterium acnes* and *Staphylococcus simulans*, as well as *Penicillium chrisogenum* that was detected in three samples (Table 4 and Fig 6).

**Table 4:** Fungal and bacterial species detected in the samples and considered as known mycetoma agents, new proposed mycetoma agents (in this study), and species detected in the samples without a clear involvement in the disease (contaminants). Numbers correspond to frequency of detection.

	Fungi		Bacteria	
**Known as mycetoma agents**	*Cladosporium ramotenellum*	1	*Streptomyces somaliensis*	1
	*Madurella mycetomatis*	4		
	*Madurella fahalii*	3		
	*Aspergillus niger*	1		
	*Aspergillus sp*	1		
	*Fusarium sp*	1		
	*Trichophyton interdigitale*	1		
**Not considered as mycetoma agents in literature**	*Penicillium thomii*	1	*Cellulosimicrobium cellulans*	2
	*Subramaniula obscura*	1	*Corynebacterium mucifaciens*	1
	*Acremonium sclerotigenum*	1		
	*Macroventuria anomochaeta*	1		
	*Subramaniula thielavioides*	1		
**Considered as contaminants**	*Penicillium chrisogenum*	3	*Staphylococcus aureus*	*5*
	*Penicillium*	1	*Staphylococcus epidermidis*	1
	*Candida parapsilosis*	1	*Staphylococcus simulans*	1
	*Malassezia restricta*	1	*Sphingomonas echinoides*	2
	*Candida albicans*	1	*Cutibacterium acnes*	1
			*Bacillus cereus*	1
			*Enterococcus malodoratus*	1
			*Klebsiella oxytoca*	1

For 14 patients (26.4%), it was not possible to detect the causative agent of the disease by any of the procedures applied.

### Treatment and patient’s outcome

Surgical removal of the mycetoma was performed in lesions smaller than 5 cm in two cases (3.5%) (Fig 2). Most of patients (40; 69%) received medical treatment as the first step, which for most of them was the only therapeutic option. Despite the problems to obtain the drugs, we were able to prescribe oral fluconazole to 28 patients (48.3%) with proven or suspected eumycetoma, and antibacterial treatment to 11 (19%) with proven or suspected actinomycetoma (7 patients were put on streptomycin plus rifampicin, and 4 patients got amikacin combined with cotrimoxazole). One patient (1.7%) received both kinds of antimicrobials (fluconazole and cotrimoxazole). In three cases (5.2%), the affected limb (foot) had to be amputated. In another patient, the big toe of he left foot was amputated before our team could evaluate the patient.

Following the course of the disease was challenging but, in the case of 11 patients (19%) it was possible to record some result through subsequent visits to the LCRH. Among the group that could be followed up on, the disease was totally healed by the treatment in three cases (27.3%) two by medical treatment and another by surgical and medical treatment. Clear improvement was found in six cases (54.5%), in terms of reduction of the affected area and function regainment. Four of these patients are still undergoing one more cycle of medical treatment. One of them referred a subsequent period of worsening because the medical treatment had to be stopped due to the COVID-19 pandemic, so the patient had to re-start fluconazole. Another patient had a bad outcome and the limb had to be amputated. For most patients (77.6%) contact was lost and no record of mycetoma status has been made so far.

Training of local professionals in the recognition and study of the disease allowed us to examine a significative number of patients for this study. Local professionals were also essential in following up some of these patients. Additionally, at least one of the members of the lab staff has now acquired the necessary skills to continue studying cultures and performing molecular biology procedures for the identification of the causative agents. In addition, most health workers at the LCRH and in the in the rural areas now know how to prevent mycetoma.

## Discussion

The occurrence of mycetoma in Kenya has been scarcely studied according to the poor information in the scientific literature. In the review by Emery & Denning (2020) [12], Kenya is mentioned as one of the African countries that on one occasion reported a high incidence of the disease, and this refers to a single article published in 1973 [15]. Subsequently, there is only one case published in 2012 by Maina and Macharia [35]. For this reason, in the analysis published by W. van de Sande *et al* in 2018, no data are provided for the disease in Kenya despite its geographical position in the so-called mycetoma belt [2]. Therefore, our study constitutes an important update of what is happening in Kenya concerning the mycetoma status, although the study only covers the Turkana County in the north of the country.

The general characteristics of the disease in Turkana are very similar to those reported in other African areas, especially in Sudan [3,6,10]. Turkana is a region neighboring South Sudan, with very similar geographic, climatological and social conditions. For the period 2013–2022, the prevalence of mycetoma in Turkana, is around 1.46. That means, very high prevalence according to the data offered by the global study conducted by van de Sande et al in 2018 [2]. The distribution inside the county shows higher prevalence in the sub-counties of Central Turkana and Loima (Table 1). This can be the effect of being closer to the LCRH where our study was centralized. Nevertheless, the mean value is high, and although the number of cases is lower in the southern part of Turkana it is still at 0.36 cases/100,000 persons/year. These numbers are in line with the mycetoma prevalence detected in Uganda, also a neighboring country of Turkana: 0.32 cases/100,000 persons/year [14].

Demographic characteristics of the disease described in our patients are in line with what has been reported before all over the world in the literature. Incidence is higher among men (73.41%), persons aged under 30 years old (48.27%) who work in the countryside, especially shepherds (51.7%) [4,6,11,36]. Like in some of these studies, we also detected a very low incidence in children [9,11,33]. Nevertheless, this data should be considered carefully as the disease has a long outcome and very frequently patients do not consult until the problem has become a cause of serious disability. At least in one of the cases collected in this study, the patient reported to have the lesion from the age of crawling (Fig 2). Other patients recorded at ages lower than 20 y.o. have mycetoma with an evolution period of more than 5 years. This would agree to what Fahal *et al* reported in a large survey in Sudan, in which young population (mainly students) were the most affected group of age [10]. Most patients had a long- standing disease at presentation and referred a mean time of evolution of their mycetoma of 6 years, with a range from 1 to more than 10 years. This is also a common characteristic of the mycetoma patients reported in many other studies [4,10]

The clinical presentation of the disease also agrees with what is reported in literature [2–7,9–12]. Most patients presented the typical triad of painless subcutaneous mass with multiple sinuses and discharge that contain grains. The foot is the most affected part of the body (87.9% of cases), followed by the hand (3 cases; 5.17%); this is widely believed to be related to the risk situations for acquiring mycetoma. The practice of barefoot walking and working in the fields barehanded predispose to traumatic inoculation of the causative organisms. In our study, hand mycetoma was detected in 3 cases, two of them, that means 13.33% of cases, detected among women (2 out of 15). Only one case was diagnosed in the hand of a man constituting 2.33% of the male cases (1 out of 43). This fact could be explained by the different professional activities developed by Turkana women and men. Men are mainly shepherds while women usually collect plant branches for building their houses (manyatas) and prepare charcoal.

Grains secretion reported or seen in most of the patients (85.2%), were mainly pale/yellow (50.8%), or black/dark (34.4%). The affected area was larger than 50 square centimeters in 57.9% of cases. Large masses have previously been associated with actinomycetoma, as they are considered to grow faster than those caused by fungi [14]. However, in our study we found no significant relationship between lesion size and the type of mycetoma (actinomycetoma or eumycetoma), nor with the color of the secreted grains, or between lesion size and time of evolution. Therefore, we believe that the specific causative agent is not the only factor determining the size and evolution of the lesion. The location in the body and the immunological status of the patient may also play a role and determine these clinical aspects.

The diagnosis of mycetoma in this series was based on the combination of clinical and laboratory techniques. Clinical diagnosis was based on the presentation and history of the disease, although most patients did not refer the onset to a trauma or thorn injury. The triad of painless slowly-growing tumor with sinuses discharging grains was the base for the clinical diagnosis. Only two (3,33%) of the selected patients were finally considered as clinically misdiagnosed. Eumycetoma was more prevalent than actinomycetoma (50% *vs* 20.3%). This distribution has been reported by other authors from different regions of Africa [3,4].

Identification methods allowed the etiological diagnosis for 21 of the 53 patients that consented samples retrieval (39.6%) which is a low rate of detection. In this regard, it should be noted that the working conditions of the samples were limited by the availability of means in the local laboratory at the LCRH. But also, because of the abrupt interruption of the work in Kenya and Spain, due to the COVID-19 pandemic lockdown in 2020. In this sense, for the 14 samples (26.4%) in which the causative agent could not be detected by any method, we consider that the biopsies were possibly not taken deeply enough or in the most optimal region of the lesion. Another aspect to be taken in account in the future is avoiding contaminants from soil and skin as much as possible.

For the mycetoma causative agents detected, the two species of the genus *Madurella* (*M*. *mycetomatis* and *M*. *fahalii*) constituted the third part of the identified agents (7/21) and were the most prevalent in our study. This finding agrees with previous reports [3,4]. Among the bacterial isolates *Streptomyces somaliensis* is the only one that was previously described as actinomycetoma agent [3,4]. Additionally, we detected *Cellulosimicrobium cellulans* in two patients showing yellow grains discharge. This genus and species had never been described before as causing mycetoma, although it has been reported as an opportunistic pathogen in immunocompromised patients [37] and causing foreign body granuloma in an immunocompetent patient that got injured by a palm thorn [38]. The onset of that reported case and the microbial characteristics of this actinobacterium, previously classified as *Nocardia cellulans* [39], led us to consider its possible involvement as the cause of actinomycetoma.

Other fungal and bacterial species detected from the samples of our patients and that had never been reported as human mycetoma agents are listed in Table 4. They were isolated from samples in which no other known causative agent could be detected. We therefore considered their possible involvement in the disease, although this needs to be demonstrated by further evidence. Some are species belonging to genera that have other members previously described as mycetoma agents, such as *Penicillium thomii* and *Acremonium sclerotigenum* each detected in one patient [40,41]. Other fungi detected in this study belong to genera not previously associated with mycetoma. One of them is the genus *Subramaniula*, of which we here describe two species potentially implicated in the mycetoma of two patients: *Subramaniula obscura* and *Subramaniula thielavioides*. This genus is considered as Chaetomiun-like fungi, phylogenetically close to *Madurella* (both belonging to Sordariales) and which have also been associated with other opportunistic superficial infections [42]. Another species reported here is *Macroventuria anamochaeta*, which has never been associated with subcutaneous pathology in mammals but belongs to the family *Didymellaceae* of the order Pleosporales, as well as the genus *Medicopsis* and others described as responsible for mycetoma cases [43].

The long distances from their homes to the LCRH without transport, combined with the onset of the COVID-19 pandemic that prevented us to visit Turkana in 2021, made it very difficult to assess the outcome of the disease in most of our patients. During this period without treatment and attention, the condition got worse in some cases. Nevertheless, among the few patients that could be followed, three (27.3%) healed and six (54.5%) showed a clear improvement, which we consider a relatively good outcome, despite the high number of the patients whose situation we were not able to assess.

Finally, as another positive result of the development of the study, it is important to mention the capacitation of the local staff of the LCRH to appropriately prevent, detect and treat the mycetoma in Turkana, despite the lack of adequate antifungals in the county.

## Conclusions

Summarizing, the findings of this put Kenya in the map of mycetoma in Africa, in the so-called *mycetoma belt*. The work offers an overview of the status of the disease in the county of Turkana, where the disease has a high incidence. Most of the clinical characteristics of the disease are very similar to what has been reported from neighboring countries such as Sudan. Some actinomycetes and fungi detected have never before been described as agents of mycetoma and need further evidence to be considered as such. In our work, no relationship was detected between time and size of the lesions and mycetoma type. This highlights the need for more studies to deeply understand the pathogenicity and the host factors that influence in the outcome of the disease. Improvement of the DNA extraction for the molecular detection of fungi involved in eumycetomas was achieved by using a modified method that could be applied in the LCRH laboratory settings. Obtention of good samples and contamination avoidance are goals to be achieved in the future.

Difficulties in providing appropriate treatment and inconveniences in the follow up of the patients, are also aspects that need special attention to improve the situation of the management of the disease in Turkana County. Education of both the rural population and healthcare workers on mycetoma is key for preventing the disease. Nowadays, there are some professionals at the LCRH that can identify the mycetoma lesions, obtain appropriate samples to study the causative agents and subsequently prescribe adequate treatment. Additionally, the public health authorities of the county are now aware of how they can prevent the spread of the disease by providing information to the most vulnerable people.

Finally, we would like to emphasize the importance of well-understood health cooperation work, in which an endemic problem is detected in the area being served, and this leads to a systematic study of the disease that can lead to a stable improvement in the health of the population targeted by the NGO campaigns.

## Supporting information

S1 Doc Patient’s data sheet. (DOCX)

S1 Fig Size of lesions in relation to: A) type of mycetoma, B) color of grains and C) time of evolution.
(PPTX)
