# Supplementary material for: First report on mycetoma in Turkana County—North-western Kenya
Source: PLoS Negl Trop Dis. 2023 Aug 14;17(8):e0011327. doi: 10.1371/journal.pntd.0011327 (PMC10449206; doi:10.1371/journal.pntd.0011327)
Supplement: S1 Doc — (DOCX) [file pntd.0011327.s001.docx]

# DATA COLLECTION SHEET

**INSTRUCTIONS**

Once the informed consent has been signed, the patient will be assigned a number that will be the result of adding the campaign code (T201X), three digits consecutively and ascending, beginning with 001. Thus, for example, the first patient which is included in the study in the February 2019 campaign is T2019 / 001. The numbering does not restart in each campaign but remains correlated for the entire project.

The microbiology team will keep the list with the identification of each patient, but only the patient code will be shown as the only identifier on the data collection form.

# MYCETOMA IN TURKANA ‐ DATA COLLECTION SHEET

PATIENT No SEX: M F

| **T** | **2** | **0** | **2** |  | **‐** |  |  |  |
| --- | --- | --- | --- | --- | --- | --- | --- | --- |

AGE (years) DATE OF SAMPLE TAKING (dd/mm/yyyy)

|  |  |  |
| --- | --- | --- |

|  |  | ‐ |  |  | ‐ |  |  |  |  |
| --- | --- | --- | --- | --- | --- | --- | --- | --- | --- |

PLACE OF BIRTH (locality / province / country):

PLACE OF RESIDENCE (locality / province / country): FROM YEAR:

PROFESSION / ACTIVITIES OF SPECIAL RISK (Agricultural activity, construction, handling of wood, ...):

TRAVELS OTSIDE TURKANA COUNTY □ YES □ NO If yes: PLACE: YEAR:

USE OF SHOES: □ YES □ NO □ OCASIONALLY KIND OF SHOES:

Have you changed your footwear because of the injury? □ YES □ NO

MODIFICATION

MYCETOMA DIAGNOSIS (mark all that apply):

1. Location of the lesion:

|  | FOOT |  | UNILATERAL: RIGHT / LEFT |
| --- | --- | --- | --- |
|  | HAND |  | BILATERAL |
|  | LEG |  | OTHER LOCATION (specify): |
|  | ARM |  |  |

1. Estimated time of evolution:
2. Size of the lesion or lesions (in centimetres):
3. Presence of secretion with grains: □ YES □ NO In case of positive COLOR of them:

Observed at the time of the exploration □ reported by the patient □

1. Previous treatment? In case of positive, please specify:
   1. How long ago?
   2. Medical treatment (If possible, specify drug, dose, schedule and duration):
   3. Surgical treatment (if possible, specify the operated area)
2. ¿Any other member of the family/community is also affected? □ YES □ NO If positive, how many? Do they live on a daily basis? □ YES □ NO
3. Does the injury prevent or modify any activity in your daily life? □ YES □ NO

If yes, specify: _

1. If it affects the foot, in relation to gait:
   1. Walks without support c. needs support (uni or bilateral)
   2. Limps d. Cannot walk

e. Other situations (specify)

SAMPLES TAKING (mark all that apply):

1. Type of sample: □ exudate □ surgical piece □ other (specify)
2. Collected as: □ swab □ tissue fragment □ aspirate □ other (specify) Sample taken by

ECOGRAFY: □ NO □ YES If Yes, date:

DATA COLLECTED BY: ………………………..……………………………………………………………………………………..

Signature
