# Supplementary material for: First report on mycetoma in Turkana County—North-western Kenya
Source: PLoS Negl Trop Dis. 2023 Aug 14;17(8):e0011327. doi: 10.1371/journal.pntd.0011327 (PMC10449206; doi:10.1371/journal.pntd.0011327)
Supplement: S1 Fig — Size of lesions in relation to: A) type of mycetoma, B) color of grains and C) time of evolution. (PPTX) [file pntd.0011327.s002.pptx]

## Slide 1
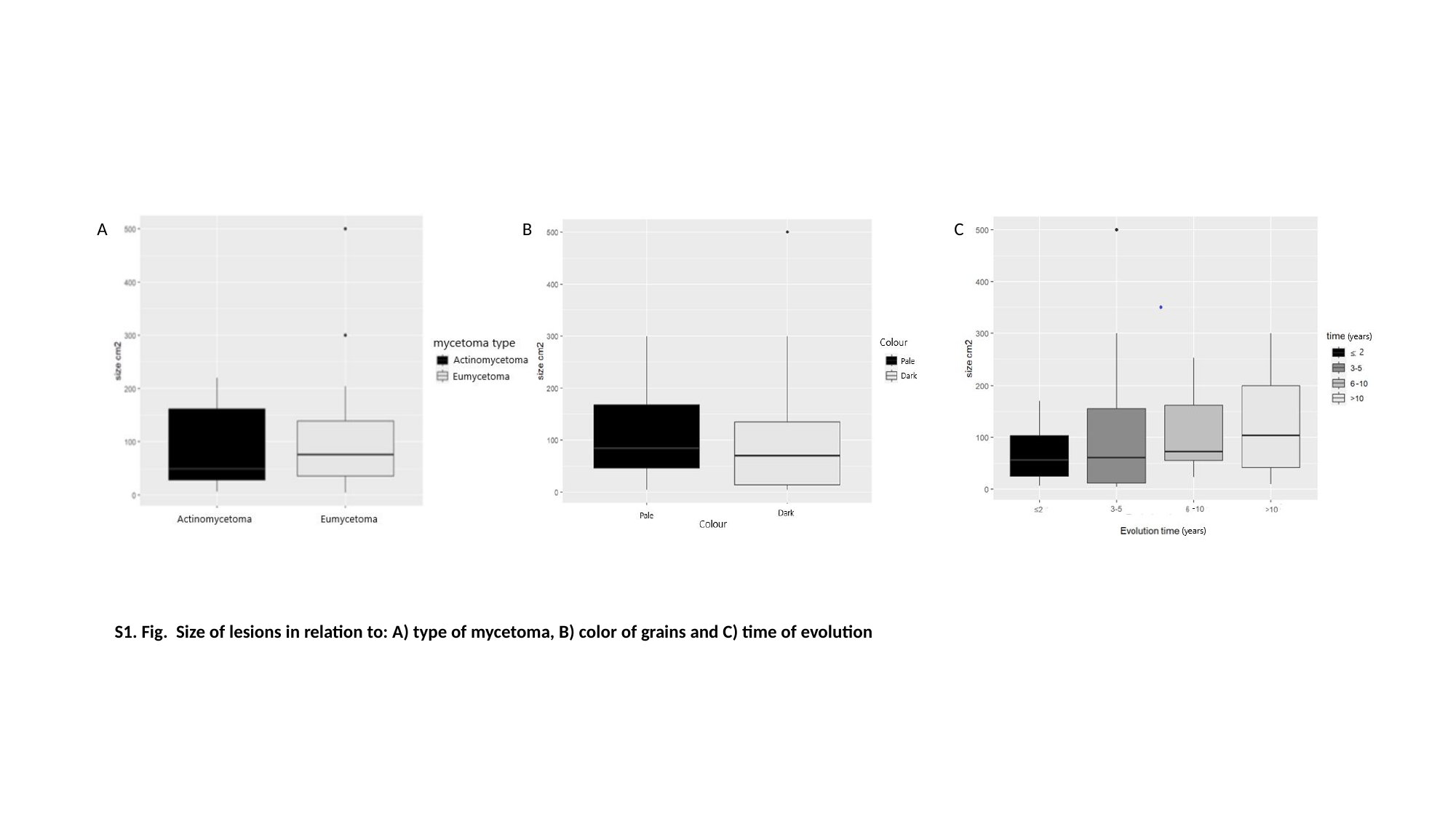

A
B
C
S1. Fig. Size of lesions in relation to: A) type of mycetoma, B) color of grains and C) time of evolution
